# Harmonization-Information Trade-Offs for Sharing Individual Participant Data in Biomedicine

**DOI:** 10.1162/99608f92.a9717b34

**Published:** 2022-07-28

**Authors:** Abel Torres-Espín, Adam R Ferguson

**Affiliations:** 1 Brain and Spinal Injury Center (BASIC), Department of Neurological Surgery, Weill Institute for Neurosciences, University of California San Francisco, San Francisco, California, United States of America; 2 San Francisco Veterans Affairs Health Care System, San Francisco, California, United States of America

**Keywords:** FAIR data sharing, data harmonization, standardization, harmonization-information trade-off, biomedicine data science

## Abstract

Biomedical practice is evidence-based. Peer-reviewed papers are the primary medium to present evidence and data-supported results to drive clinical practice. However, it could be argued that scientific literature does not contain data, but rather narratives about and summaries of data. Meta-analyses of published literature may produce biased conclusions due to the lack of transparency in data collection, publication bias, and inaccessibility to the data underlying a publication (‘dark data’). Co-analysis of pooled data at the level of individual research participants can offer higher levels of evidence, but this requires that researchers share raw individual participant data (IPD). FAIR (findable, accessible, interoperable, and reusable) data governance principles aim to guide data lifecycle management by providing a framework for actionable data sharing. Here we discuss the implications of FAIR for data harmonization, an essential step for pooling data for IPD analysis. We describe the harmonization-information trade-off, which states that the level of granularity in harmonizing data determines the amount of information lost. Finally, we discuss a framework for managing the trade-off and the levels of harmonization. In the coming era of funder mandates for data sharing, research communities that effectively manage data harmonization will be empowered to harness big data and advanced analytics such as machine learning and artificial intelligence tools, leading to stunning new discoveries that augment our understanding of diseases and their treatments. By elevating scientific data to the status of a first-class citizen of the scientific enterprise, there is strong potential for biomedicine to transition from a narrative publication product orientation to a modern data-driven enterprise where data itself is viewed as a primary work product of biomedical research.

## Media Summary

The goal of biomedical research is to produce evidence to understand, prevent, and treat diseases. Doing so requires that scientific data are accurate, available, and generalizable enough to support reliable decision-making in medical practice. Biomedical data are judged by the imperfect process of scientific peer review and published literature rather than raw data sets. Typically, studies are evaluated based on summaries and conclusions, and the raw data from individual research participants remains inaccessible. Literature-based summaries are subject to biases and author interpretations and can mask information hidden in the raw data. Thus, to maximize the return from funding the biomedical research enterprise (estimated at U.S. $240 billion in 2009, worldwide) data must be shared. To promote this, the U.S. National Institutes of Health (NIH) has recently announced their 2023 data-sharing mandate that adheres to the FAIR (findable, accessible, interoperable, reusable) data stewardship principles, guiding researchers, institutions, and agencies to elevate scientific data to 'first-class citizen' status as a product of research. The authors discuss how making data FAIR will strengthen the evidence for medical practice by facilitating the reuse of data from different data sets, an important step in analyzing independent studies together. FAIR requires harmonization to ensure fused data elements convey the same information, producing interoperability. This article articulates the trade-offs that researchers must make during the harmonization process, balancing the level of harmonization of data sets against the level of information lost in doing so. Finally, the authors discuss a framework to help manage the information loss and to increase the potential for harmonization across shared data, readying them for emerging applications of machine learning and artificial intelligence in support of higher levels of evidence in biomedicine.

## 1. Introduction

Data sharing in biomedicine has emerged as one of the solutions to improve scientific transparency and reproducibility. The National Institutes of Health (NIH) 2023 Data Management and Sharing Policy will accelerate these efforts with the goal of increasing the value of federally funded research and reducing waste by providing direct access to data for replication and pooled individual participant level (IPD) analysis (Chan et al., 2014; Ferguson et al., 2014; Kennedy, 2012; Office of the Director, NIH, 2020; Piwowar et al., 2007; Pronk et al., 2015; Roundtable on Environmental Health Sciences et al., 2016). To help implement data-sharing practice, the National Academies of Sciences, Engineering, and Medicine (NASEM) held a series of meetings 2019–2021 with diverse stakeholders across funders, universities, libraries, technologists, and researchers from biomedicine and social sciences ("Changing the Culture," 2021; NASEM 2020a, 2020b). The present *HDSR* special theme titled “Changing the Culture on Data Management and Data Sharing in Biomedicine” focuses on changing the culture of data sharing to facilitate uptake of data sharing at a grassroots level in scientific communities. In this review, we address features of biomedical data collection and management practices that limit the harmonization, integration, and pooling of data in biomedical research communities. Our goal is to articulate current cultural norms within biomedicine/biological research with respect to data sharing and to discuss structural problems that limit the implementation of data sharing that maximizes its usability (Callahan et al., 2017; Chan et al., 2014; “Changing the Culture,” 2021; Fouad et al., 2019; NASEM, 2020a; Torres-Espín, Almeid, et al., 2021). We argue that researchers make a series of compromises from the point of raw data collection through to reporting of results in scientific papers and the potential reuse of that data if shared. The use of data formatting and collection standards such as Clinical Data Interchange Standards Consortium (CDISC, 2022) data standards or NIH Common Data Elements (NIH CDE, 2022) can help support data FAIRness (Wilkinson et al., 2016), although they might not be sufficient in their own. Implementation of industry-grade standards is being advanced by initiatives like the Coalition for Accelerating Standards and Therapies (CFAST, 2022), a partnership with CDISC and the Critical Path Institute for the development of standards for therapeutic areas of interest such as Alzheimer's disease (Critical Path for Alzheimer’s Disease; Sivakumaran et al., 2020). Yet, the widespread use of data standards in biomedical research is still a need. And even when studies are designed to implement standards, interoperability and reusability can break down at several steps of the data lifecycle (Kush et al., 2020). Acknowledging and formalizing the intermediary steps in the path from raw to literature-reported data has potential to improve data-sharing practice for biomedical researchers, clinicians, journals, universities, funders, and the general public whose tax dollars support scientific discovery.

In this article, we discuss data sharing throughout the biomedical data lifecycle. The article is divided into five sections. Section 2 introduces the problems of publication bias and data inaccessibility and the threats they cause for high-quality evidence and bench-to-bedside translation of scientific findings. We present open sharing of IPD as a possible solution. Section 3 introduces the issue of data granularity, a balance between increasing sample size and the number of features collected in biomedicine, and its implications for IPD harmonization. Section 4 offers an overview of best practices to promote interoperability and reusability and improve the harmonization of IPD. Section 5 conceptualizes the loss of information that occurs when harmonizing IPD as a harmonization-information trade-off that should be actively managed. Section 6 concludes with a summary. Box 1 provides operational definitions for the terms used throughout.

## 2. Publication Bias, Its Effect on Levels of Evidence, and the Need for Data Access

Current medical practice is evidence-based, requiring scientific publications be weighed and synthesized by expert committees according to levels of evidence ranking systems prior to translating biomedical research into the clinical practice (Burns et al., 2011; Canadian Task Force on the Periodic Health Examination, 1979; Guyatt et al., 1992). For regulated medical interventions, the Food and Drug Administration (FDA) requires submission of raw data using specific data standards (FDA, 2021). However, outside of this specific regulatory context, decisions to implement medical interventions rely on published evidence synthesis and guidelines derived from the available published literature. Meta-analysis is considered the top of the pyramid of levels of evidence, establishing the gold standard for medical implementation of scientific findings (Burns et al., 2011; Debray et al., 2015; Glass, 1976, 2000). Classic meta-analysis is carried out by aggregating summaries and descriptive statistics from numerous studies, usually extracted by systematic review of peer-reviewed publications, which assumes that all available studies provide sufficient evidence for a scientific finding (Glass, 1976; McNamara & Scales, 2011). However, literature-based meta-analysis has several drawbacks, including the fact that only published papers and reports are considered in levels of evidence ranking systems. This fails to account for the fact that the published literature represents a small fraction of the total data collected by the biomedical research enterprise. Moreover, the small fraction of data published in manuscripts may reflect a highly biased subset of the data that happen to support the hypotheses of the authors, enabling them to tell a strong enough story about their findings to survive peer review. This is known as ‘publication bias’ or the ‘file-drawer phenomenon’ whereby only large effects appear in the peer-reviewed literature, and results with smaller effects are relegated to file drawers within faculty offices around the world.

Metanalyses have suggested that publication bias violates a common scientific assumption that the peer-reviewed published literature provides a representative sample of findings from all studies, including unpublished studies, conducted on a topic (Scargle, 1999; Sterling et al., 1995). Sterling et al. make a compelling argument through a systematic review suggesting that 20% of studies should demonstrate the null effect hypothesis, yet the published literature show a much lower proportion of null findings (Sterling et al., 1995). Meta-analytic techniques (funnel plots and egger regression) (Duval & Tweedie, 2000; Peters, 2006) and systematic reviews on the topic demonstrate that effect sizes in the published literature are substantially skewed toward large effects, with the largest effects being seen in studies with the smallest sample sizes and lowest power, suggesting that many reported ‘large effects’ in the literature may actually reflect random noise in statistical distributions of effect sizes (Sena et al., 2010; Sterling et al., 1995; Watzlawick et al., 2014, 2019). By imputing missing small-effect sizes to restore expected normal distributions of effect sizes, it is possible to quantify the degree to which the published literature overestimates true effect sizes (Duval & Tweedie, 2000; Sena et al., 2010; Watzlawick et al., 2019). Beyond biased effect sizes, published papers leave out important information required to accurately gauge results, as has been exemplified in meta-analyses comparing peer-reviewed publications versus unpublished clinical study reports from the same trials (Doshi et al., 2012). Ioannidis (2005) and others have argued that the published literature in biomedicine reflects prevailing biases rather than generalizable findings (Holman et al., 2016; Ioannidis, 2005; Sena et al., 2010; Watzlawick et al., 2019). Support for this idea comes from recent reports that biomedicine has a reproducibility crisis, and that most large effects in the published literature cannot be independently replicated (Baker, 2016). In this context, literature-based meta-analysis and the levels of evidence ranking systems in biomedical research may fall prey to the endemic problems of publication bias.

Inaccessible data (‘dark data’) is estimated to comprise between 30% to 50% of the data collected by the biomedical research (Chan et al., 2014; Galsworthy et al., 2012; Scherer et al., 2018). This is a major contributor to estimates that 85% of the biomedical research investment worldwide is wasted (Chalmers & Glasziou, 2009). To solve the problems of publication bias and inaccessible data, we and others have argued in favor of transparent data sharing at the individual participant level (Chan et al., 2014; Ferguson et al., 2014; Macleod et al., 2014), independent of how ‘publishable’ a study is. Individual participant data meta-analysis seeks to mitigate some of the issues of traditional meta-analysis by pooling raw data from individual research subjects on a large scale instead of relying solely on data extracted from published reports. Sharing data across research projects for IPD meta-analysis allows for more robust analysis, circumventing publication bias if data is systematically accessible (Burke et al., 2017; Debray et al., 2015; Riley et al., 2010; Thomas et al., 2014). The first step in IPD co-analysis of data from separate studies is to integrate or harmonize distinct data sets, ensuring the comparability of measures across studies. For effective pooled IPD analysis, data from different studies must be findable and accessible independent of their chance for manuscript publication and have sufficient detail and documentation about data collection and data format. This documentation itself must be obtained for each study to control for biases, confounding variables, and sources of heterogeneity in subsequent meta-analysis. In addition, most biomedical research studies are statistically underpowered due to small sample sizes (Button et al., 2013; Dumas-Mallet et al., 2017). Pooled IPD can overcome underpowered studies by increasing sample sizes beyond that of individual studies to resolve robust effects from a family of similar studies (Riley et al., 2010, 2020). However, determining the similarity of different studies requires interpretation and reporting of variables collected, and becomes a problem of data harmonization that we will cover in greater detail in Section 3.

## 3. Data Granularity and Harmonization

**Figure 1.**
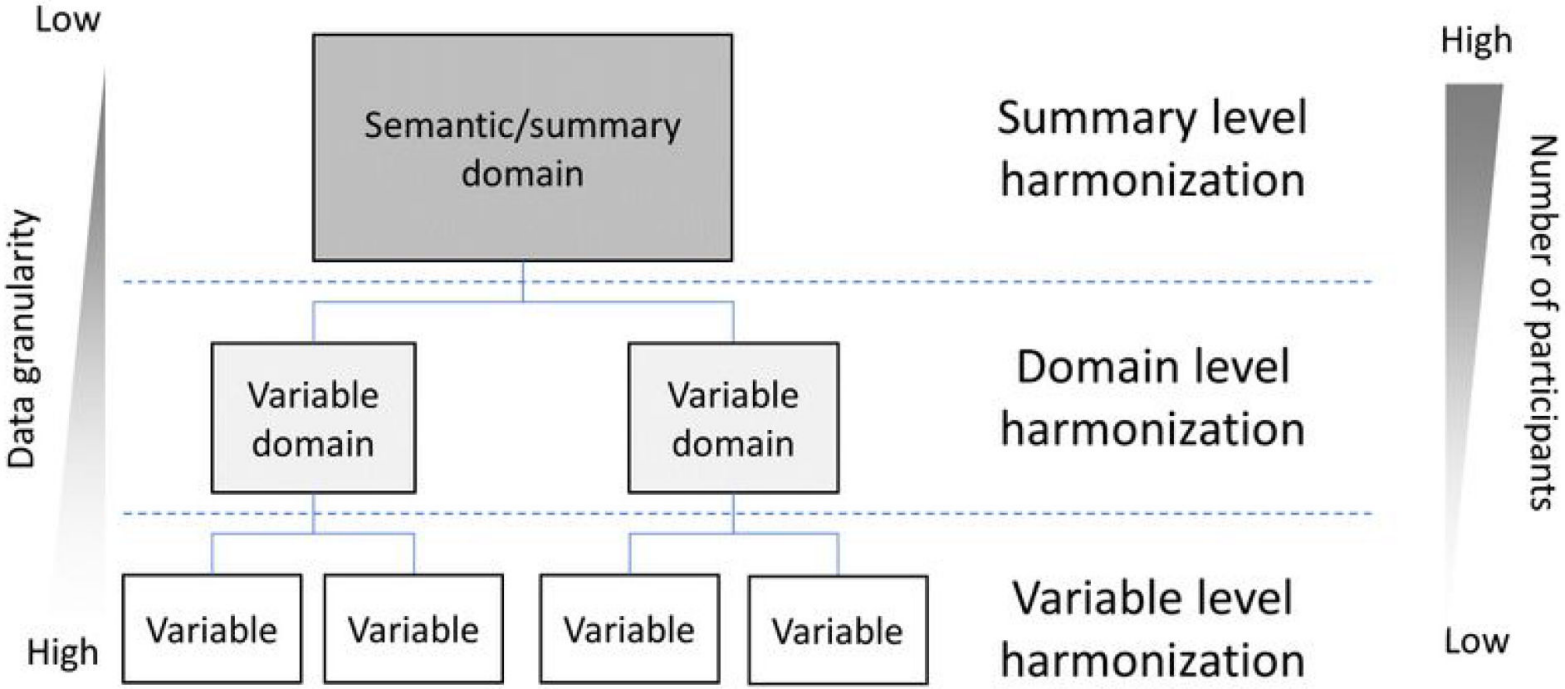
Levels of data granularity at different harmonization goals. The more granular and deep the harmonization is (at the single variable/participant level), the more information is available. In pooled analysis, deeper harmonization often comes at the cost of reducing the number of participants that can be potentially harmonized. Harmonizing at the level of aggregate measures or summaries that represent a semantic domain (e.g., memory deficits) can increase the number of subjects in a harmonized data set since there may exist several ways to measure that same semantic domain. As we dive deeper into the variable levels, finding methods for accurate harmonization can be more challenging, and lower numbers of subjects may be available in the harmonized data set.

Every decision made during data collection and data sharing affects data reusers’ ability to harmonize data sets, and ultimately to derive high-level evidence for accelerating biomedical research and medical implementation. This can be thought of as a trade-off between summary knowledge reported in scientific literature and information contained in granular data at the level of individual variables and participants (Figure 1). An illustrative example comes from traumatic brain injury (TBI) studies, where functional tests are commonly performed to evaluate different subject’s neurological ability (Nelson et al., 2017). For instance, verbal learning and memory can be tested through the California Verbal Learning Test (CVLT) or Rey Auditory Verbal Learning Test (RAVLT). Although similar, these two tests are not interchangeable (Stallings et al., 1995). These tests usually have three levels of synthesis: the level of the individual item (i.e., values of each test and question performed), the level of the domain that groups of items represent (e.g. Attention span, Learning efficiency, Delayed recall, Inaccurate recall)(Wiegner & Donders, 1999), and at the level of summary scores derived from all items to describe subject’s performance in a single metric. Two TBI studies performing one of these tests each could be harmonized at the level of the single summary score, which captures the semantic meaning of the test (e.g., learning and memory), at the level of the variable domain, or at the individual item level. In general, it would be easier for studies to find ways to harmonize at the common semantic level of two tests that are designed to measure the same concept, which we refer to as ‘shallow harmonization,’ rather than domain or item levels, which we call ‘deep harmonization.’ In biomedicine this often translates into a trade-off in the number of research participants available for analysis and the number of harmonized variables (Naselaris et al., 2021), as well as the accuracy of the harmonization (Griffith et al., 2013). Because epidemiological approaches emphasize high sample sizes to boost statistical power, the biomedical literature is filled with large but information-poor (shallow) data sets that feature a small number of variables with high numbers of subjects. In the emerging fields of digital health and precision medicine, the emphasis is on gaining an information-rich (deep) data set capturing a more detailed picture of each individual subject. This results in high granularity in multiple variables, but generally low numbers of participants who are deeply phenotyped using multidimensional disease features (Naselaris et al., 2021). Efforts such as the NIH All of Us million-person precision health study are poised to generate deeply harmonized data that are both high volume and high variety, leading to unprecedented big data that are both information rich and high in sample size (Lyles et al., 2018; “‘All of Us’ Research Program,” 2019). A few other studies such as the Framingham Heart Study, the Alzheimer’s Disease Neuroimaging Initiative (ADNI), and Transforming Research and Clinical Knowledge in Traumatic Brain Injury (TRACK-TBI) are notable examples of data that are both big in volume and wide in variety (Andersson et al., 2019; Donohue et al., 2017; Huie et al., 2018; Jack et al., 2013; Kannel et al., 1972; Mahmood et al., 2014; Yue et al., 2019; Yuh et al., 2021). The authors have direct experience with two of these major efforts (TRACK-TBI and ADNI), and our perspectives in the current article have been influenced by our roles as data scientists working in this area. Harmonization for advanced IPD analysis is not a foregone conclusion even in these large-scale prospective studies. Attention to the harmonization granularity required for analysis provides a critical roadmap for sharing data across centers and studies. In general, more granular harmonization makes data more Artificial Intelligence (AI)-ready by providing rich features for these data-hungry analytic methods.

The FAIR data principles (Wilkinson et al., 2016) provide an important framework to guide data-sharing processes across these levels of granularity, stating that scientific data must be findable, accessible, interoperable, and reusable. The first two principles are relatively easy to accomplish in today’s data-sharing landscape, as the plethora of sharing repositories expands. However, the interoperability and reusability of data are more difficult to achieve as they require both a cultural uptake by the data collectors, as well as the development of tools, policies, standards, and infrastructures, beyond simple access to the data (Fouad et al., 2019; Kush et al., 2020; Nielson et al., 2014; Torres-Espín, Almeida, et al., 2021). Therefore, during the journey from initial data collection to data sharing, several compromises must be made by different stakeholders, affecting the harmonizability, interoperability, and reusability of the shared data, and ultimately our ability to conduct pooled IPD analysis. While interoperability means that data can be integrated, it does not ensure that the information and meaning of the pooled data resources are sufficiently similar for analysis, which is achieved through data harmonization. The following sections will explain our understanding of the relationship between interoperability, reusability, and harmonizability. We advocate for FAIR and harmonizable data (FAIR+H) to accelerate medical implementation.

## 4. Interoperability, Reusability, and Harmonizability

Data harmonization requires a systematic process similar to systematic literature reviews for validity and robustness (Fortier et al., 2017). Recommendations, guidelines, and tools have been developed to harmonize data from studies with distinct designs. When the studies do not follow exact same standards across all variables, we can consider the process of harmonization as ‘retrospective harmonization’ (Fortier et al., 2017). When studies are designed to maximize harmonization and integration from the beginning of study conceptualization, it is known as ‘prospective harmonization’ (Fortier et al., 2017; Hicks et al., 2013; Meeuws et al., 2020). In reality, there is a continuum between full retrospective to full prospective harmonization, in which different standards, interoperability, and reusability practices may affect the ease of harmonization.

### 4.1. Retrospective Harmonization

Retrospective harmonization requires studies considered for harmonization share enough similarity in their data collection that information across data sets is “inferentially equivalent,” meaning that original variables or derived variables during the process of harmonization convey the same information, regardless of differences in measurement methods (Fortier et al., 2011, 2017). The qualification of equivalence varies depending on the nature of the data and the subject of study (Fortier et al., 2017; Griffith et al., 2013; Kalter et al., 2019). However, a general consideration is to find balance between only considering strictly equivalent variables (i.e. that have been collected using the same specifications, methods, tools, and constraints) on the one hand, and providing some flexibility in considering data across diverse data collection mechanisms (Kalter et al., 2019) on the other. The more heterogeneous a research field is in their data collection process, the more difficult retrospective harmonization becomes, reducing the chances for inferential equivalence. For example, in the field of traumatic brain injury, researchers collect brain images, clinical features, neuropsychological evaluations, and molecular biomarkers on the same subject. This results in extreme heterogeneity in variable formats even within the same study. In the preclinical literature, there is even wider heterogeneity, with different laboratories collecting entirely different subsets of measures using homegrown methods and customized assessment tools. A few examples of heroic data recovery from dark data records do exist, but these efforts typically take both deep domain knowledge and uncommon tenacity to convert raw data into an interoperable format for IPD analysis. For example, Marmarou et al. (2007) harmonized data from over 11 clinical trials into a single database to develop the IMPACT prognostic model for traumatic brain injury (Marmarou et al., 2007; Steyerberg et al., 2008). Similarly, researchers in the spinal cord injury field recovered and digitized 20-year-old data from a multicenter animal spinal cord injury study and deployed modern machine intelligence tools to discover new predictors of neurological recovery (Almeida et al., 2021; Nielson et al., 2015), that were later successfully translated into clinical studies (Torres-Espín, Haefeli, et al., 2021). However, these undertakings required years of targeted effort and funding that are unlikely to scale. Designing studies at the outset for future data harmonization and integration provides an attractive alternative to retrospective harmonization.

### 4.2. Harmonizable by Design, Standards, and Prospective Harmonization

Prospective harmonization considers data sharing as part of the study design process by adopting standard methods for data formatting, definition, and collection (Box 2). Prospective application of standards improves equivalence across studies (Fortier et al., 2017; Hicks et al., 2013; Meeuws et al., 2020), facilitating the process of data integration and harmonization. While the use of standards is common in clinical trials, and recommendations for the sharing of IDP data from such studies have been suggested (Ohmann et al., 2017), adoption of data-sharing standards in small laboratory studies are rare. Ideally, standards should include three components to ensure maximal reusability and painless harmonization: 1) a common data models (CDM) specifying formatting to increase interoperability; 2) common definitions and representations (i.e., terminologies, vocabularies, coding schemes); and 3) standard procedures for data collection. An example of a common data model is the Observational Medical Outcome Partnership (OMOP) CDM (Overhage et al., 2012; Stang et al., 2010), which provides a standard for interoperable formatting in relation to specific standardized medical vocabularies. Several other CDMs exist, such as i2b2 (Deshmukh et al., 2009), PCORNet (2022), and CDISC CDM standards (CDISC, 2022). Mapping algorithms that ensure data format interoperability between these CDMs have been developed (Klann et al., 2016, 2019). Even then, further harmonization may be needed (Haendel et al., 2021). One example of a common vocabulary and data collection standards is the U.S. National Institutes of Neurological Diseases and Stroke (NINDS) common data elements (CDEs), a set of well-defined variables and examples of data collection tools (clinical research forms or CRFs)(Biering-Sørensen et al., 2015; Hicks et al., 2013; LaPlaca et al., 2021; Meeuws et al., 2020). To date, NINDS CDEs been developed for 21 disorders including stroke, epilepsy, traumatic brain injury, spinal cord injury, among others (NINDS, 2022). Designing and collecting data that implements CDEs, reduces the barrier for downstream data harmonization. Yet, CDEs only provide the semantics that facilitate variable interpretation and ‘inferential equivalence.’They do not provide standards for data formatting and structure, limiting interoperability and harmonization (Kush et al., 2020). This creates sources of variation introduced in the process of study execution such as site-specific database schemas, data collection tools (e.g., custom CRFs), data cleaning practices, data improvements, and knowledge-based annotations during the point of data reuse. These require attention during the harmonization process. On the other hand, the use of CDM without proper common representations or semantic data collection standards such as CDEs would facilitate the digital joining of data but fail to provide assurance on the inferential equivalence across data sets. These issues must be actively managed throughout the data lifecycle to ensure continued interoperability, reuse, and harmonization of biomedical data sets. Considering FAIR practices at the point of study design, before data collection, can greatly reduce the cost and effort of downstream sharing and increase the interoperability, reusability, and harmonizability of shared data (Box 2).

One must consider that even when data is collected and organized under some standards, pooling data from sources using different standards may require harmonization. An example is the NIH National Center of Advancing Translational Science (NCATS) National COVID Cohort Collaborative (N3C) initiative (covid.cd2h.org). N3C systematically and regularly collects data derived from the electronic health records for the study of COVID-19 (Haendel et al., 2021). Different medical institutions and health care organizations provide data sets in four different CDMs. In order to ingest and integrate the data, the N3C data harmonization team developed a workflow to harmonize definitions and transform all four CDMs to a common one (OMOP). Quoting their work “Simply aggregating those data together is insufficient. Not only does each model have different structures and values, but heterogeneity exists within models” (Haendel et al., 2021, p. 433). The harmonization workflow was conducted over several review meetings and with the presence of subject matter experts from the source data. This work illustrates that even in situations where data might be collected under robust standards such as CDMs, new research questions may require further harmonization. Other examples come from the neuroimaging field, where multisite projects such as ADNI (Jack et al., 2008; Petersen et al., 2010), the Human Connectome Project (HCP) (Glasser et al., 2013; Van Essen et al., 2012), and the Adolescent Brain Cognitive Development (ABCD) study (Bjork et al., 2017) were prospectively designed with data sharing in mind, yet pooling data still required additional work. Overall, although the use of standards by design substantially reduces the effort of data pooling, additional harmonization may still be needed depending on the goals and objectives at the point of data reuse.

It should be noted that legal and ethical challenges may also affect the ability to perform prospective harmonization at the IPD level. For example, in neuroscience research, a field with an increasing volume of shared data from small and large projects, differences in international and state laws threaten data sharing, pooling, and reuse. This has triggered efforts to define international data governance for neuroscience (Eke et al., 2021) that could be adopted and generalized to other fields. The NIH 2023 data-sharing policy will also likely spur development of new legal frameworks to assist in design of prospective harmonization and data management policies.

## 5. The Information Lost

A classic practice for data harmonization in biomedicine is to start by defining a hypothesis, a narrow scientific question, and then selecting which data sets and specific variables require harmonization to answer the specific question at hand (Fortier et al., 2017). This approach ensures robust harmonization by focusing on a small and manageable set of variables. However, with the ever-increasing data resources and computational capabilities, high volumes of data are becoming available for data-intensive analytics such as machine learning, that do not necessarily conform to hypothesis-driven investigation (Huie et al., 2018; Margolis et al., 2014; Obermeyer & Emanuel, 2016). In addition, with the rise of precision medicine and omics-based clinical studies, biomedicine is moving away from narrow hypothesis-driven questions and increasingly toward data-driven-discovery that is broad and information rich. Therefore, different scientific questions may be asked using the same list of data sets, but they may require different levels of harmonization. For instance, consider the problem of age-related degenerative diseases. An epidemiology researcher could build a clinical prediction model from IPD metadata with a small set of desired covariates such as age, brain volume, and cognitive decline by narrowly harmonizing data from multiple publicly available data sets such as those made available through ADNI (Petersen et al., 2010). On the other hand, a digital health researcher with the goal of deeply phenotyping brain degeneration with complete electronic health records, wearable smartwatch monitors, and multi-omics (genome, transcriptome, proteome, metabolome) would benefit from harmonizing as many variables as possible on each patient across studies. Therefore, data harmonization efforts to answer hypothesis-driven vs. data-driven questions have a different scope and may need different approaches. A researcher may harmonize the data to answer a hypothesis-driven question and then later perform a new harmonization for asking the second question. In practice, there are a dizzying number of potential questions and approaches that could be considered and applied to the same harmonized data set, and predicting all of them at the point of data sharing is near-impossible. Having to reharmonize the same data sources every time a new question is to be tested (i.e., incorporation of new variables) is tedious and time consuming. An alternative is a tiered framework for dealing with different levels of data granularity that may conform with different harmonization needs. This requires expanding the harmonization task from an afterthought to a flexible and living harmonization process that may be particularly productive for biomedical research consortia.

### 5.1. Harmonization-Information (H-I) Trade-off

**Figure 2.**
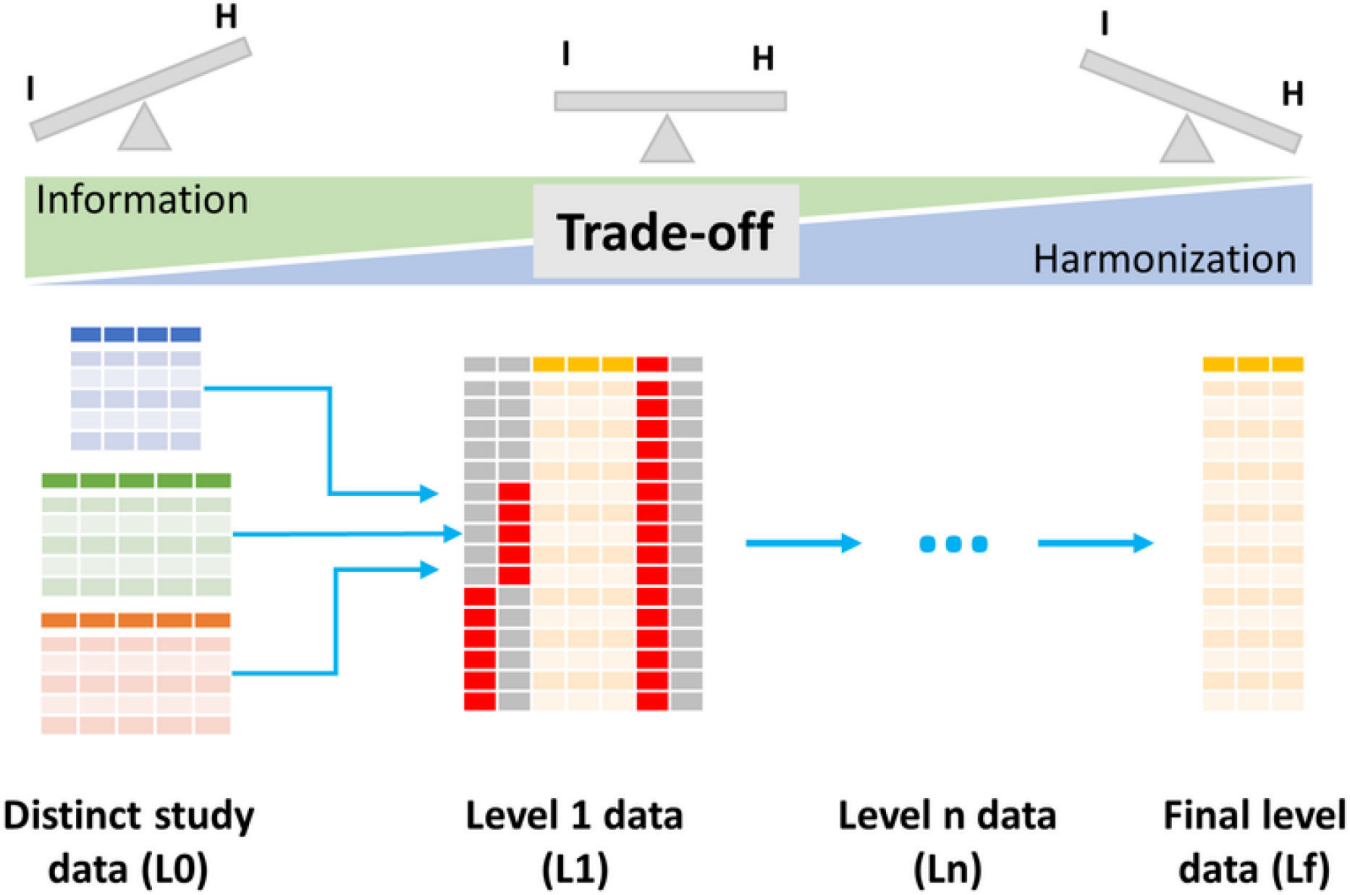
The harmonization-information trade-off can be managed through a tiered system of increasing harmonization, allowing for flexibility to choose different levels of the trade-off depending on the analytical goal. At the L0 level of the trade-off distinct data sets to be pooled are represented by different colors. At the L1 level the data sets are combined to produce a pooled data set where orange variables represent those that can be pooled without harmonization, harmonizable values with transformation are represented in grey, and mismatches are represented in red (not harmonizable). Through transformations (e.g., changing in scale, finding a set of minimal common categories, binning) different levels of harmonization (L2, L3, …, Ln) can be achieved prior to arrival at the final data harmonization for analysis (Lf). The number of levels in this workflow might depend on study goals and complexity of harmonization.

In this section we describe the Harmonization-information (H-I) trade-off encountered when pooling data across studies (Figure 2). In the following section we describe a tiered system for managing harmonization, with clear-eyed acknowledgment that IPD data pooling requires compromises to maximize both the number of subjects and the number of harmonized data elements used in analysis. During the harmonization process, there is a potential loss of information in derivative data sets, depending on the degree of similarity (e.g., number of harmonizable variables) between the different data sets. Our goal is to provide a practical approach for prioritizing variables for data harmonization to enable pooled data reuse for data-driven and hypothesis-driven questions.

Let us consider a simple example of a demographic variable such as the level of education of participants. A study in Europe and another in the United States may ask participants the same question, ‘What is your highest level of education?’ Semantically, these two studies are collecting the same information in the same way, however, given international differences in education systems, harmonizing this variable between studies may require finding a common ground of lower information (e.g., binning granular levels of education into ‘primary,’ ‘secondary,’ ‘postsecondary’), or developing rules to infer bins of years of education. No matter which transformation is applied, the new harmonized variable will contain less granular information than the original ones. This constitutes a trade-off between the level of harmonization we target, and the amount of information lost. Ideally, two perfectly matching data sets would not need harmonization, and would not lose information when pooled together. This rare situation would not require any effort other than recoding variable names in cases where naming conventions differ across countries of origin. In practice, there will always be a choice between retaining the maximal information from the original set of data versus gaining the advantages of a harmonized data element for pooled analysis.

For example, in a study of cardiovascular disease we may determine that rescaling continuous numerical variables, such as height, blood pressure, and walking speed, into z-scores is an acceptable loss of information (losing the original scale for each variable), if it allows us to pool data across hospitals for analysis. However, compressing a 15-point ordinal neurological coma score collected in one hospital into a two-category (alive, dead) score collected at another hospital may be too much of an information loss for our purposes, and therefore we would decide to drop these variables from the harmonization, thereby excluding this information from all downstream analyses. Establishing the level of information loss that one is willing to trade for harmonization provides a strategy for developing tiered data products for use in subsequent analyses.

### 5.2. Managing the H-I Trade-Off With Data Harmonization Levels

To manage the H-I trade-off, we recommend that biomedical researchers plan their FAIR data curation around data harmonization levels (Figure 2, Box 3), analogous to the data-processing levels that NASA uses for earth-observing satellite data (EarthData, n.d., earthobservatory.nasa.gov). The lowest level of harmonization (L0) contains the maximal information possible and consists of the original collective set of data sets considered for pooling. The next levels are defined by different grades of harmonization with increasing transformation, and therefore greater information loss. For example, L1 data might consist of joined data from all L0 components, pooling data for all those variables that are identical across L0, and maintaining the remaining variables untransformed or annotated as noncollected (coding for ‘missingness’) for each data element. The next level (L2) builds from L1 by performing the next set of defined transformations on those variables that data were not pooled in the previous level but that can be harmonized across data sets. This sequence proceeds through as many steps as required until all harmonizable variables are harmonized (including new derived variables if required), obtaining intermediate levels of H-I trade-off with increasing loss in information as data sets become more harmonized. Maintaining separate study data sets in isolation will allow for zero information loss but also zero harmonization of variables across data sets (Figure 2, L0). On the other extreme, keeping only equivalent variables (that do not need harmonization) across data sets with no required transformation produces a pooled data set, but at the expense of losing most of the information by dropping most of the variables (Figure 2, Lf). In the middle (Figure 2, L1, L2, L3, etc.) we find a wide range of harmonization levels, depending on the amount of transformation we are willing to accept for each variable.

**Figure 3.**
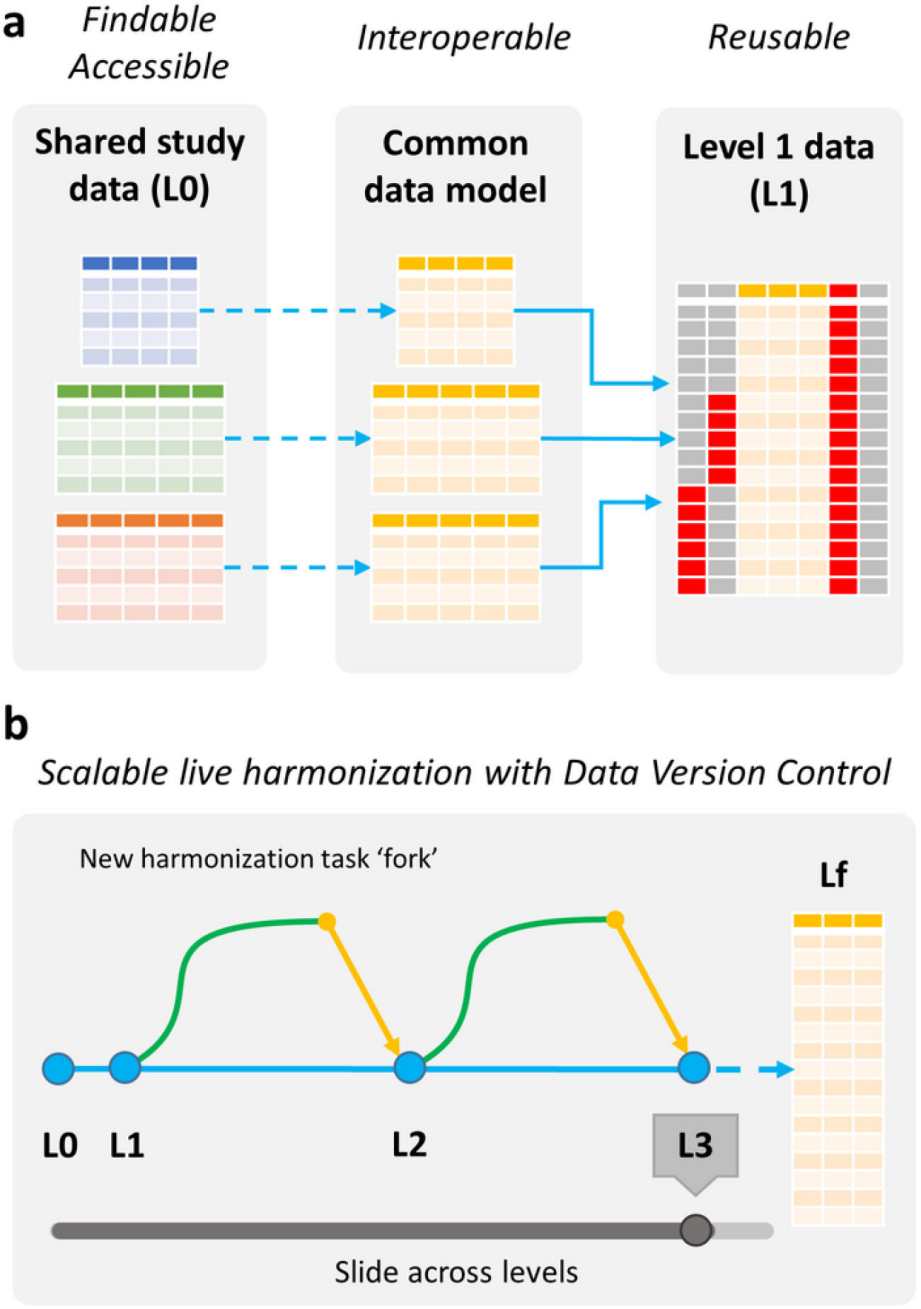
FAIR principles through a live harmonization. Adopting FAIR improves harmonization **(a)**. Designing studies for interoperability reduces the cost and time of data formatting to a common model, and using common vocabulary during data collection ensures higher alignment and reusability of the data sets. Harmonization through data version control **(b)**. It is possible to traverse or slide across levels of harmonization from raw data to fully harmonized derivative data sets. The data lifecycle from the point of collection through to analysis can be viewed in a version control context as a series of ‘forks’ in data harmonization as data are readied for reuse in a diverse set of pooled analysis contexts.

In practice, performing several of these incremental steps might be unworkable or unnecessary, although this might be at the discretion of the harmonization team. In our own efforts we have found that L1 to L3 of these intermediate steps are reasonable. The final level (Lf) data constitutes the most harmonized data set, with the maximal level of information loss we are willing to consider. Each harmonization step can be fully automated using open source software, and harmonization code itself can be made FAIR and publicly available to ensure reproducibility. If done using a version control system for data such as Git, it is possible to arbitrarily traverse across levels of harmonization from raw data to fully harmonized pooled data sets. In this way, the data lifecycle from the point of collection through to analysis can be viewed in a version control context as a series of ‘forks’ in new data harmonization tasks as data are readied for reuse in a diverse set of analysis contexts (Figure 3). For example, DataLad (Halchenko et al., 2021), a free open source distributed data management system that builds on Git can be used to capture data transformations that track data provenance through the lifecycle, enabling automatic computation and reproducible data harmonization and pooling.

## 6. Conclusion

In this article we have discussed practical issues for FAIR data reuse, data harmonization, and analytics for biomedicine. We have argued that emerging data sharing policies such as the NIH 2023 policy are likely to result in more actionable insights if research communities take on the problem of data standardization and harmonization as a flexible and scalable framework. We conceive of this as productionized workflow for scientific data where raw data materials are taken in and processed into harmonized derivative data products, enabling a wide variety of potential reuses and analysis workflows. Understanding data refinement as a trade-off between information content and harmonization level has potential to allow researchers to flexibly manage the data lifecycle from the point of data collection of individual variables through to large-scale knowledge discovery through analysis and semantic workflows. By elevating scientific data to the status of a first-class citizen of the scientific enterprise there is strong potential for biomedicine to transition from a narrative publication product orientation to a modern data-driven enterprise where data itself is viewed as a primary work product of biomedical research. The 2023 mandate is poised to accelerate discovery and lead to new types of scientific careers, especially for young scientists who are digital natives and are comfortable traversing the boundaries of the harmonization-information trade-off. The transition is likely to create shockwaves in biomedical research communities. However, research communities that effectively manage data harmonization will be able to harness the energy of this shock with machine learning and artificial intelligence tools, leading to stunning new discoveries that augment our understanding of diseases and their treatments.

## Box 1. Operational Definitions

**Table:**

**Data:** Factual information (e.g., measures) collected in a study that is the basis for scientific claims or assertions (e.g., narratives about data or papers). The more granular the features (i.e., the greater the number of possible values or states of a variable), the more information contained by the data.
**Variable:** Measure, quantity, or element that defines a set of data collected in a study.
**Individual Participant Data (IPD):** Data that is available at the level of the individual participant (human) or subject (animal) in a study, constituting the smallest independent unit of analysis. IPD data is used to produce aggregated summary measures for groups of subjects (e.g., mean; standard deviation).
**Data Sharing:** The process by which data are made available to others for (re)use. Manuscript publication and statistical reports are not considered Data, but rather narratives about and summaries of data.
**FAIR:** Stewardship principles that state that data must be findable, accessible, interoperable, and reusable, providing a guided framework on how to share data on the Web (Wilkinson et al., 2016).
**Standards and standardization:** Data standards are rules and specifications to assure consistency and regularity in collection of data. Standardization is the process of conforming data to concrete standards.
**Data harmonization:** The process by which data from different sources and studies are transformed to make them as comparable as possible, with the goal of integrating them together. This may include changes in naming conventions, statistical transformations, data reformatting, semantic crosswalking, among others.
**Data integration or pooling:** The act of combining data from different sources that are deemed comparable, either after the results of data harmonization, or by a priori study design. We refer to pooling and integration interchangeably.
**Semantic interoperability:** The capacity to integrate data that have the same meaning. A variable collected in different studies that has the same meaning across studies is said to have semantic interoperability. Common terminologies and vocabulary can set standards for semantic interoperability.
**Data format interoperability:** The capacity to exchange data because they are formatted in the same way or in forms that can be interchanged (mapped to each other). Format interoperability can be promoted using the same formatting standard. Note, format interoperability does not guarantee semantic interoperability.

## Box 2. Increasing FAIRness and Harmonizability of Shared Data

**Table:**

Study stage	Recommendations for increasing FAIRness and harmonizability of shared data
**Design**	**Choose a data format with common data model (CDM) compatibility.** While designing the experiment, a data format that adheres to formatting standards would facilitate to share the data under a common data model. Software transforming data from one format to another through a common data model can reduce the cost of harmonization by automating the data extraction, transform, load (ETL) process required for integrating different datasets. **Choose a standard vocabulary and measures.** Collecting data under semantic standards allow for better reusability of the data, facilitating the process of variable alignment and increasing ‘inferential equivalence’ between studies to harmonize. Considering vocabulary standards in the field of study during the design phase would improve the value of the shared data and increase the pool of studies that the research community can reuse. **Choose standard procedures and protocols.** The same metrics can be collected with variation even under the same standard vocabulary if the procedures, protocols, and tools for data collection are different. Determining standard operating procedures (SOP) common in a field of study can reduce deviations introduced during data collection.
**Data Collection and Curation**	**Minimize variations in protocols, data entry, and data management tools.** Document any changes made in data values, data format, vocabularies, and protocols. This information facilitates identification of potential inconsistencies during harmonization. Using data version control systems can reduce the documentation effort.
**Data Sharing**	**Release documentation together with the data.** Data dictionaries or codebooks are necessary for documenting the meaning of the data to promote reusability and downstream harmonization efforts. The use of standard vocabularies can facilitate data dictionaries. If the study data is formatted with compatibility to a CDM, providing the documentation, and scripts if available, would reduce the barrier for interoperability. Documentation should also include protocols, SOPs, data collection tools, as well as potential deviations from these.

## Box 3. Levels of Data Harmonization

**Table:**

**Level 0 (L0):** Collection of raw data sets accessible for each shared study. At this level there is no harmonization beyond the one produced by design, and data sets may be presented in different formats, files, schemas, and so on, as different software are used to collect and store the original data. This level contains the maximal information possible among the considered data sets.
**Level 1 (L1):** This data set contains a combination of all the variables from the original data sets after formatting to a common data model (CDM). Harmonization is attempted at face value, aligning variable name of those variables and instruments that might be comparable by design. For instance, demographics and standardized measures are likely to be pooled at this level. At this stage, no variables are dropped, producing a sparse data set. This allows for new data sets to be incorporated in future efforts. Codifying for missingness such as ‘not collected’ would provide further information on potential harmonization steps in subsequent levels. This is the minimal level of harmonization feasible, with no loss of information.
**Level n (Ln):** Subsequent levels build on the previous level (L2 from L1) by considering further transformations of the data such as aggregation to common denominator or statistical transformations, or deriving new harmonized variables as needed. As transformations are produced, the level of harmonization increases at the expense of losing information.
**Level final (Lf):** The final level of harmonization is reached when no more transformations are possible and the nonharmonizable variables are dropped. Theoretically, there are different points at which the process can be considered at the maximal level of harmonization (i.e., new derivative variables are almost always possible). Practically, a researcher may want to set a criterion beyond which no further harmonization is attempted. The maximal harmonization level is achieved when only the equivalent variables are considered, producing the maximal loss of information.
